# Idiopathic Spontaneous Occurrence of Heterotrophic Occurrence During Pregnancy

**DOI:** 10.5435/JAAOSGlobal-D-22-00204

**Published:** 2023-01-18

**Authors:** Christina Melian, Aadit Shah, Emaan Alvi, Brendan Boyce, Fazel Khan, James Penna

**Affiliations:** From the Renaissance School of Medicine at Stony Brook University, Stony Brook, NY (Dr. Melian); the Department of Orthopaedic Surgery (Dr. Shah, Dr. Khan, and Dr. Penna) and the Department of Pathology (Dr. Alvi and Dr. Boyce), Stony Brook University Hospital, Stony Brook, NY.

## Abstract

Heterotopic ossification (HO) typically presents in the hip, knee, and elbow joints in the setting of trauma or postsurgical intervention. Less commonly, it may occur secondary to neurologic dysfunction or underlying genetic conditions, but idiopathic HO is rare. Most cases of HO are managed nonoperatively with surgical resection remaining a controversy due to high recurrence rates. We describe a case of idiopathic HO of the shoulder that occurred in the absence of trauma, neurologic dysfunction, or underlying genetic disorder that was treated with surgical excision.

Heterotopic ossification (HO) is the formation of ectopic bone within the soft tissues.^1^ HO can be classified into three broad categories based on etiology: traumatic HO from fractures, dislocations, orthopaedic surgeries, and burns; neurogenic HO from spinal cord damage or head injury; and genetic HO from conditions, such as progressive fibrodysplasia ossificans or progressive osseous heteroplasia.^2^ Traumatic HO comprises up to 90% of cases, most commonly a complication of arthroplasty or bone fracture, with a higher predisposition for men.^3^ Although it can occur anywhere in the body, HO typically affects areas most susceptible to trauma, such as the elbow, thigh, pelvis, and shoulder.^4^

When symptomatic, HO can present with pain, decreased range of motion, and in severe cases, bony ankylosis.^2^ The diagnosis is made with radiographic imaging, including radiographs, CT, and MRI. Several classification systems have been used to grade the severity of HO, but the Brooker Classification System used for HO at the hip^5^ and the Hastings and Graham System for HO at the elbow^6^ are two of the most commonly used.

A definitive diagnosis of HO can be made by histopathologic evaluation of a tissue sample. HO is often defined pathologically by the type of tissue in which it occurs, such as myositis ossificans involving skeletal muscle. The histopathologic features of the lesion typically evolve over time, with early lesions being composed of cellular fibrovascular tissue with small amounts of bone matrix. More mature lesions have prominent bone formation with a zonal architecture, a hallmark of HO. It is important to differentiate HO from a neoplastic process, such as soft-tissue sarcoma or osteosarcoma. Microscopically, in more mature lesions, trabeculae of woven bone with osteoblastic rimming accompanied by more mature lamellar bone is more consistent with HO, rather than osteosarcoma. The treatment of clinically significant HO is surgical resection. Milder forms may be managed conservatively with NSAIDs and physical therapy.^2^

In the absence of trauma, neurologic dysfunction, or a genetic disorder, HO is rare. We report a case of HO of the shoulder presenting as abrupt onset pain in the setting of pregnancy, without any preceding trauma, neurologic dysfunction, or genetic etiology.

## Case Report

A 29-year-old woman at 15 weeks of gestation presented to our emergency department with excruciating right shoulder pain. She initially presented to an outside hospital after 3 weeks of similar shoulder pain aggravated by any movement. She was given oxycodone with no pain relief. She reported a medical history of excision of a melanoma of the left arm but was otherwise in good health. Her pregnancy had been uncomplicated, and she did not have any recent traumatic injuries. Physical examination demonstrated diffuse tenderness to palpation about the right shoulder, upper arm, latissimus dorsi, and neck. Passive range of motion of the right shoulder was 160° of forward flexion (FF), 40° of neutral external rotation (ER), and 85° of abduction compared with 170° of FF, 50° of neutral ER, and 85° of abduction in the left shoulder. Active range of motion of the right shoulder was 140° of FF and 45° of neutral ER compared with 170° of FF and 45° of neutral ER in the left shoulder. A positive Neer Impingement sign and Hawkins sign were observed on the right. No signs of shoulder instability were noted.

**Figure 1:**
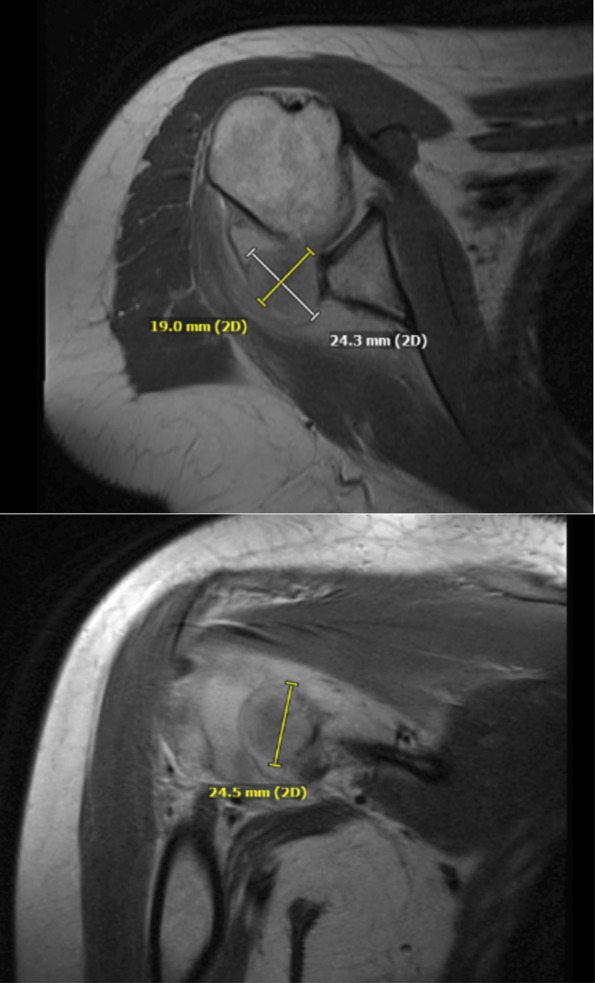
MRI showing lobulated intermediate hyperintense mass within the posterior glenohumeral recess.

MRI of right shoulder revealed a lobulated intermediate hyperintense mass within the posterior glenohumeral joint recess abutting the posterior glenoid and labrum (Figure 1). Moderate pericapsular-free fluid and edema were seen within the belly of the adjacent teres minor muscle. No rotator cuff or labral tears were observed. Mild tenosynovitis of the long head of the biceps was noted. The CT scan was deferred because of pregnancy.

Ultrasound-guided core needle biopsy of the soft-tissue mass was done, and histologic examination revealed cores of fibrovascular myxoid tissue, with areas of prominent osteoid formation, prominent blood vessels, and focal mild chronic inflammation consistent with HO. Tissue culture grew *Staphylococcus lugdunensis* that was isolated in the broth only, likely a contaminant. Laboratory findings included a white blood cell of 13.07, erythrocyte sedimentation rate 28, CRP 0.8. Given the patient's notable discomfort, surgery was indicated. Radiation therapy and NSAID use were discounted because of pregnancy.

**Figure 2:**
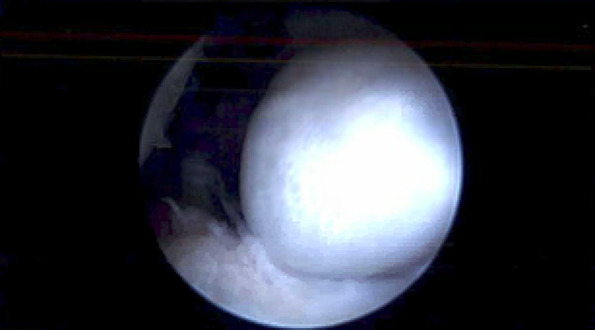
Intraoperative arthroscopy showing calcified ovoid mass.

**Figure 3:**
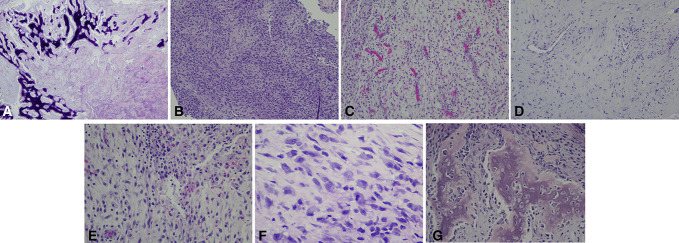
**A**, Extensive osteoid production with peripheral calcium pyrophosphate calcification, H&E stain, 20x magnification. **B**, Cellular fibrous stroma with a swirling pattern, H&E stain, 100x magnification. **C**, Loose connective tissue stroma with interspersed vessels and focal areas of acute inflammation, H&E stain, 100x magnification. **D**, Myxoid stroma containing benign fibroblasts, H&E stain, 100x magnification. **E**, Loose connective tissue stroma with focal areas of acute inflammation, H&E stain, 200x magnification. **F**, Rare mitotic figure in fibromyxoid stroma, H&E stain, 400x magnification. **G**, Osteoid production with interspersed fibromyxoid stroma, H&E stain, 200x magnification.

The patient underwent a right shoulder arthroscopy with excision of the mass (Figure 2). Pathologic examination of the excised tissue (Figure 3) showed a fibromyxoid and osseous lesion, with features consistent with HO. The combination of benign osteoblast proliferation with extensive osteoid production, and fibromyxoid proliferation with benign spindle cells, with focal peripheral calcification best fit an early phase of HO.

The patient was seen in clinic 2 days after surgery with marked improvement in pain. The use of a sling was discontinued, and she was started on physical therapy and Tylenol, as needed for pain. The patient was seen in the orthopaedic clinic 2 weeks later with full recovery. Informed consent was obtained before publication.

## Discussion

Our patient presented with a 3-week history of acute onset, severe shoulder pain, and limited range of motion in the absence of trauma, neurologic dysfunction, or genetic predisposition. With careful history, and physical and radiological evaluation, the possibility of this being an intra-articular neoplasm was explored but ultimately led to the diagnosis of HO. One of the most common precipitating factors of HO in adults is trauma.^2,3,4,7^ Traumatic HO is a consequence of abnormal wound healing, systemic and local immune system activation, infection, extensive vascularization, and innervation.^8^ It occurs most frequently as a postsurgical complication, with up to 40% of cases arising from hip arthroplasty and up to 30% from fractures or dislocation of the elbow.^4^ Surgical factors, such as extended ischemia time, type of approach, and the use of cemented implants, have been shown to increase the risk of traumatic HO after arthroplasty.^3^

Although less common, neurologic injury and genetic conditions may also predispose an individual to HO. Up to 50% of spinal cord injuries result in neurogenic HO,^4^ other less common neurologic conditions, such as meningitis and brain tumors, have been implicated in the development of neurogenic HO.^2^ Genetic HO is rare and develops because of conditions, such as fibro dysplasia ossificans progressive (fibrodysplasia ossificans), progressive osseous heteroplasia, and Albright hereditary osteodystrophy. Such genetic disorders typically begin in childhood and present with multiple areas of HO.^2^

In rare cases, idiopathic HO may occur without a precipitating insult or underlying genetic condition.^9,10^ Our case report demonstrates the development of idiopathic HO of the shoulder in the setting of pregnancy. Although HO can form anywhere in the body, the most affected areas include the hip, knee, and elbow.^7,11^ HO of the shoulder is rare, even in the setting of arthroscopic surgery^7^; when present, it is typically asymptomatic and not clinically significant.^11^ To the best of our knowledge, 26 cases of nontraumatic HO of the shoulder have been described in English literature: nine infectious,^12,13,14,15,16,17,18,19^ six genetic,^20,21,22,23,24,25^ six neurologic,^26,27,28,29,30,31^ three autoimmune,^32,33,34^ one drug-induced,^35^ and one idiopathic.^10^

Although the etiology of HO is diverse, the pathophysiology is thought to be the same across all subtypes. Three components have been recognized to contribute to the development of HO: pluripotential cells, molecular signals for differentiation, and a suitable microenvironment for bone formation.^36^ The release of bone morphogenetic (bone morphogenetic) from normal bone in response to venous stasis, inflammation, or connective tissue disease has been shown to play a role in the differentiation of pluripotential cells into osteoblasts.^9,36,37,38^ Prostaglandin E2 likely also plays a role in the differentiation of progenitor cells.^9,37^ Studies have shown that a hypoxic microenvironment promotes chondrogenesis, thereby playing a key pathogenetic role in the early stages of HO.^9,38^

A diagnosis of HO can be made with conventional radiographs or CT; however, out of an abundance of caution, given this patient's pregnancy status, MRI was used because it has been shown to have similar efficacy while providing less radiation exposure.^39,40^ Asymptomatic patients can be managed conservatively with NSAIDs and physical therapy.^4,7,41^ In patients with clinically significant HO who present with severe pain and limited range of motion, as seen in our patient, surgical excision is indicated.^38^ Studies have shown notable postoperative improvement in clinical and functional outcomes after surgical resection of HO.^7,10,42^ Arthroscopic removal of the ectopic bone is believed to be the best approach because it allows for excellent visualization of the subacromial space and acromioclavicular joint while causing minimal soft-tissue trauma.^7,10,42^ Although arthroscopy is a well-known risk factor for the development of HO, prophylaxis with NSAIDs and low-dose radiation therapy have been shown to reduce the recurrence of HO after surgical removal.^9,10,41^ Excision of ectopic bone before 6 months has been associated with an increased risk of HO recurrence,^4^ and surgeons should carefully consider these factors when excision is indicated. Early rehabilitation is recommended postoperatively to maximize outcomes and prevent HO recurrence.^7^

## Conclusion

We present an unusual case of shoulder HO in the absence of trauma, neurologic dysfunction, or genetic predisposition. When planning arthroscopic excision, orthopaedic surgeons should consider the functional impairment caused by HO with the increased risk of surgical complications, such as damage to neurovascular structures and HO recurrence. Optimal medical management is also imperative in preventing HO recurrence and regaining functional abilities.
